# Factors Related to Family Caregivers Quitting Their Work

**DOI:** 10.1093/geroni/igaa057.238

**Published:** 2020-12-16

**Authors:** Masakazu Shirasawa, Yoshihito Takemoto, Kazutaka Masuda, Ryousuke Hata

**Affiliations:** 1 International university of Health and Welfare, Nabari, Mie, Japan; 2 Okayama Prefectural University,, Soujya, Okayama, Japan; 3 Mukogawa Women’s University, Nishinomiya, Hyogo, Japan; 4 Hokusei Gakuen University,, Sapporo, Hokkaido, Japan

## Abstract

As the working population declines in Japan, preventing family caregivers from quitting their work has become a government priority. Approximately 100,000 people leave their jobs annually because of caregiving obligations. The present study examines the reasons behind caregivers’ resignation and the factors that prevent caregivers from quitting. In January 2018, 3,000 sites were randomly selected from care management institutions nationwide. Surveys were conducted by mail, first with one care manager from each institution, then with an elderly person requiring long-term care and who had a family caregiver overseen by that same manager. The second survey was contingent on the response to the first. A total of 1,719 valid responses were received in the first survey (response rate: 57.3%), and 594 in the second survey (response rate: 34.6%). The surveys found that 21.2% of family caregivers quit their jobs. Caregivers also quit their hobbies (23.6%), neighborhood associations (7.2%), and stopped volunteer activities (5.4% ). Eight items from the survey of people requiring long-term care and who were supported by their family caregivers, and 5 items from the survey of care managers were analyzed in binomial logistic regression analysis with continuation of work (yes/no) as the dependent variable. Caregivers are less likely to continue working if they are older and their dependents require extended care, and more likely to continue working if they and their dependents are satisfied with the care manager. Care managers could therefore play a crucial role in allowing caregivers to find a better balance between caregiving and work.

